# Intestinal Microbiota Regulate Xenobiotic Metabolism in the Liver

**DOI:** 10.1371/journal.pone.0006958

**Published:** 2009-09-09

**Authors:** Britta Björkholm, Chek Mei Bok, Annelie Lundin, Joseph Rafter, Martin Lloyd Hibberd, Sven Pettersson

**Affiliations:** 1 Department of Microbiology, Tumor and Cell biology, Karolinska Institutet, Stockholm, Sweden; 2 Genome Institute of Singapore, Singapore, Singapore; 3 Department of Biosciences and Nutrition, Karolinska University Hospital, Huddinge, Novum, Stockholm, Sweden; Charité-Universitätsmedizin Berlin, Germany

## Abstract

**Background:**

The liver is the central organ for xenobiotic metabolism (XM) and is regulated by nuclear receptors such as CAR and PXR, which control the metabolism of drugs. Here we report that gut microbiota influences liver gene expression and alters xenobiotic metabolism in animals exposed to barbiturates.

**Principal findings:**

By comparing hepatic gene expression on microarrays from germfree (GF) and conventionally-raised mice (SPF), we identified a cluster of 112 differentially expressed target genes predominantly connected to xenobiotic metabolism and pathways inhibiting RXR function. These findings were functionally validated by exposing GF and SPF mice to pentobarbital which confirmed that xenobiotic metabolism in GF mice is significantly more efficient (shorter time of anesthesia) when compared to the SPF group.

**Conclusion:**

Our data demonstrate that gut microbiota modulates hepatic gene expression and function by altering its xenobiotic response to drugs without direct contact with the liver.

## Introduction

Data accumulated over the last decade strongly support that the commensal intestinal flora has profound effects on the physiology of the host. In the majority of studies to date, these effects have been studied locally in the intestine, where members of the microbiota are known to affect angiogenesis, development of the intestinal epithelium as well as the mucosa associated lymphoid tissue (MALT) [1]–[4]. However, more recent work is indicating that the flora may be exerting effects beyond the intestine and studies have indicated that patients with some chronic inflammatory disorders such as obesity, Type I diabetes and Crohn's disease display an altered gut flora that may be influencing the phenotype of these disorders [5]–[8].

The liver is an essential organ in mammals and the central organ for metabolic processes including xenobiotic metabolism. Moreover, the liver also overlooks and tunes homeostasis of cholesterol, bile acids, sterols, lipids and heme. Nutrients and metabolites are transported from the intestine to the liver via the afferent pathways, the vena portae and the lymphatic system. In contrast, the efferent bile ducts transport molecules from the liver to the intestine thereby closing the loop and giving rise to the enterohepatic circulation system. Thus, a vast array of metabolites circulates between the intestine and liver. Hence, the enterohepatic circulation is a critical transport system in order to allow the liver to play its central role in mammalian metabolism. Previous data have shown that intestinal microbes can tune the profile of lipids in the enterohepatic circuit thus having consequences for liver metabolism [9]–[12]. The direct effects of microorganisms on the cholesterol-derived compounds and their hepatic recirculation renders hepatic metabolism a plausible systemic physiological function regulated by intestinal microbiota, without direct contact with the bacteria.

Xenobiotic metabolism in the liver is largely regulated by nuclear receptors (NRs), primarily the “xenosensors” Constitutive Active/Androstane Receptor (CAR), and the Pregnane X receptor (PXR) [13]. In addition, CAR and PXR also regulate homeostasis of cholesterol, bile acids, sterols, lipids, heme and other endogenous hydrophobic molecules. They form a NR network regulating a number of essential liver functions jointly with the bile acid activated Farnesoid X receptor (FXR) and the liver X receptor (LXR) [13]. The cross-reactivity of lipid homeostasis and xenobiotic metabolism pathways was recently pin-pointed to converge at the transcription factor Insig-1 being regulated via both CAR and PXR [14]. This report elegantly provided an explanation as to how an activation of xenobiotic metabolism regulators like CAR and PXR induce Insig-1 by CAR and PXR which may explain the negative effects of some drugs on hepatic lipid levels [14]. Thus, the pathways for xenobiotic metabolism and homeostasis of sterols, lipids, bile acids, heme and energy cross-react in the liver through NRs [14], [15].

Recently, exploiting germfree (GF) animals, we reported that several NRs (including CAR) in the colonic epithelium are subjected to regulation by the microbiota [16]. The effects of a complete intestinal microbiota on liver metabolites and xenobiotic compounds have been studied since the 1960ies. However, this field has been largely neglected in the recent decades. Motivated by this and with the overall aim of gaining insight into potential molecular mechanisms by which the intestinal microbiota might influence the metabolic potential of the liver, we undertook an investigation of the molecular effects of a normal intestinal microflora on the liver and its function. Our results show that the microbiota have extensive effects on hepatic gene expression in mice, demonstrated by higher expression of a number of key genes in metabolic function, most notably CAR, in GF mice as compared to conventionally-raised specific pathogen-free (SPF) animals. This effect is most likely a result of microbial metabolism of endogenous compounds in the intestine. The physiological effects of the gene expression results were confirmed by examining the metabolism of the xenobiotic pentobarbital, in GF and SPF mice. Our results connect the intestinal bacteria to xenobiotic metabolism in the liver and supply a potential mechanism for this effect.

## Results

### Effect of intestinal microbiota on gene expression in liver

**Figure 1:**
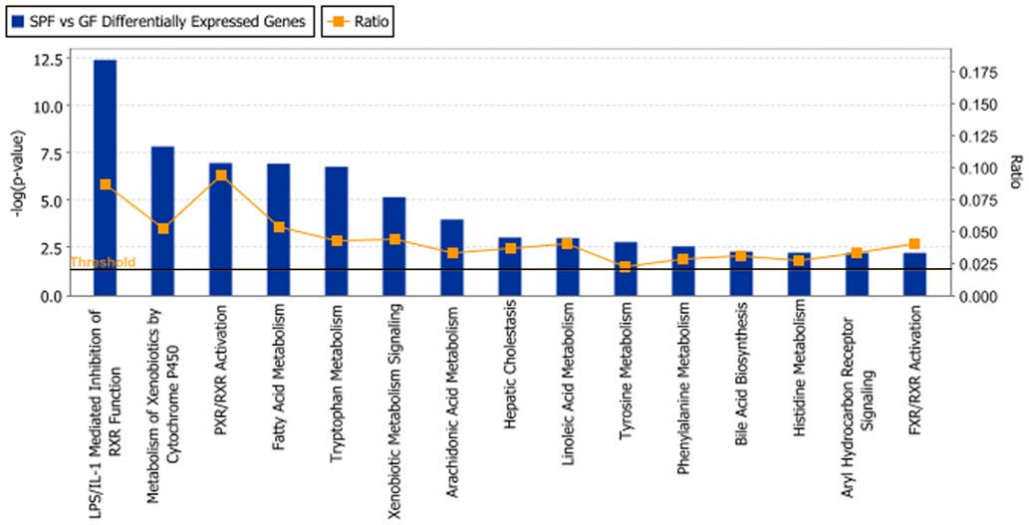
Canonical Pathways Analysis. Shown are the top fifteen significant canonical pathways as determined by using the Ingenuity Pathway Analysis software (Ingenuity® Systems). The strength of the statistical association is indicated by the length of the bars. The ration value reflects the proportion of gene elements in the differentially abundant gene list that corresponded to genes in each pathway. The vertical line represents the threshold of the significance.

**Figure 2:**
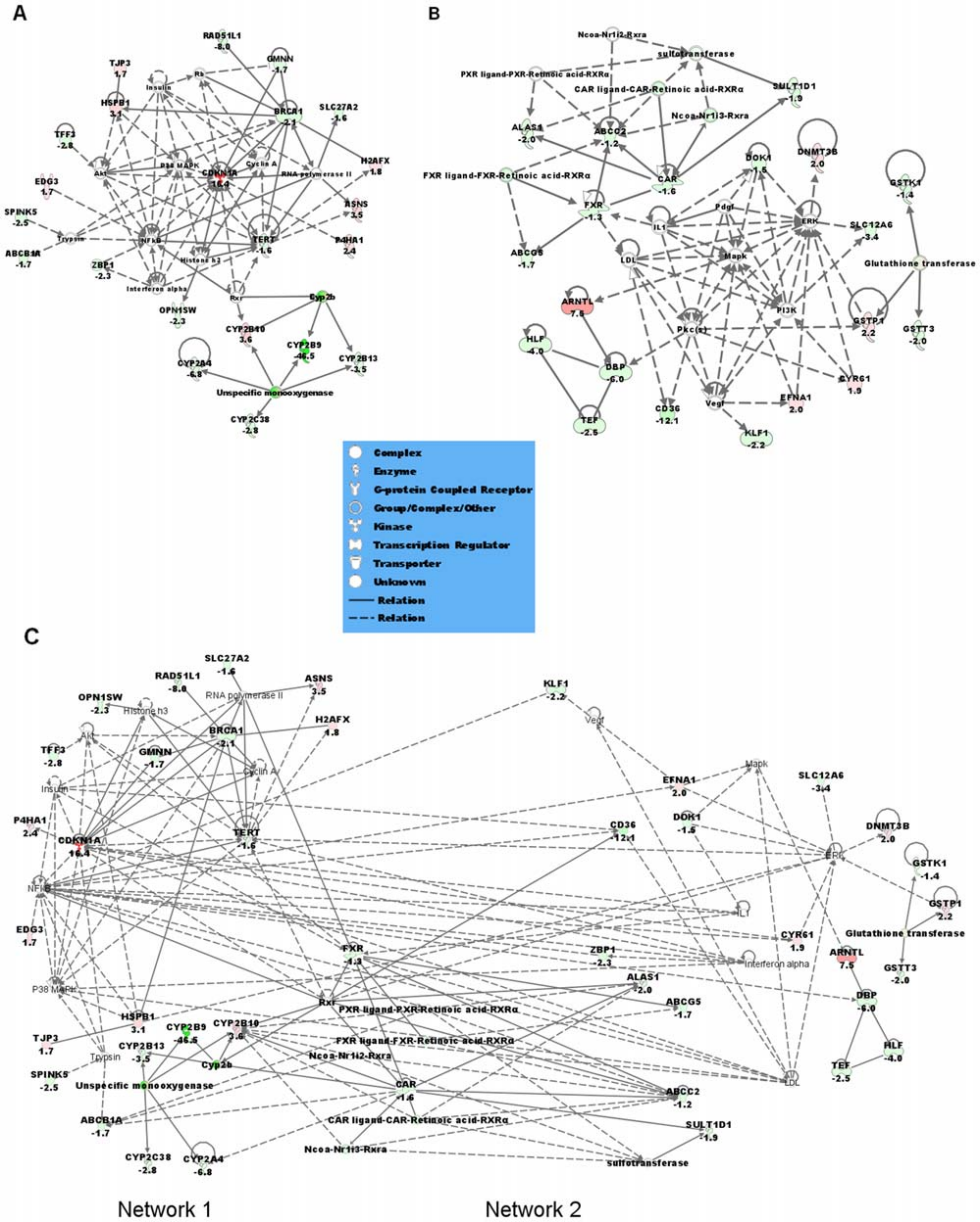
Pathway analysis based on the Ingenuity Pathway Knowledge Base (IPKB). The two highest scoring networks (A) Network 1 and (B) Network 2 created from the 112 differentially expressed genes list are shown. (C) showed the merged network of Network 1 and Network 2. Nodes that are not colored are added by IPKB. Colored nodes are shaded by their relative expression, green when lower in GF than SPF and red when higher expressed in GF compared to SPF, intensity is relative to expression. The shape of the node indicates the major function of the protein. A line denotes binding of the products of the two genes while a line with an arrow denotes ‘acts on’. A dotted line denotes an indirect interaction. Yellow lines in (C) denotes connection between Network 1 and Network 2.

**Figure 3:**
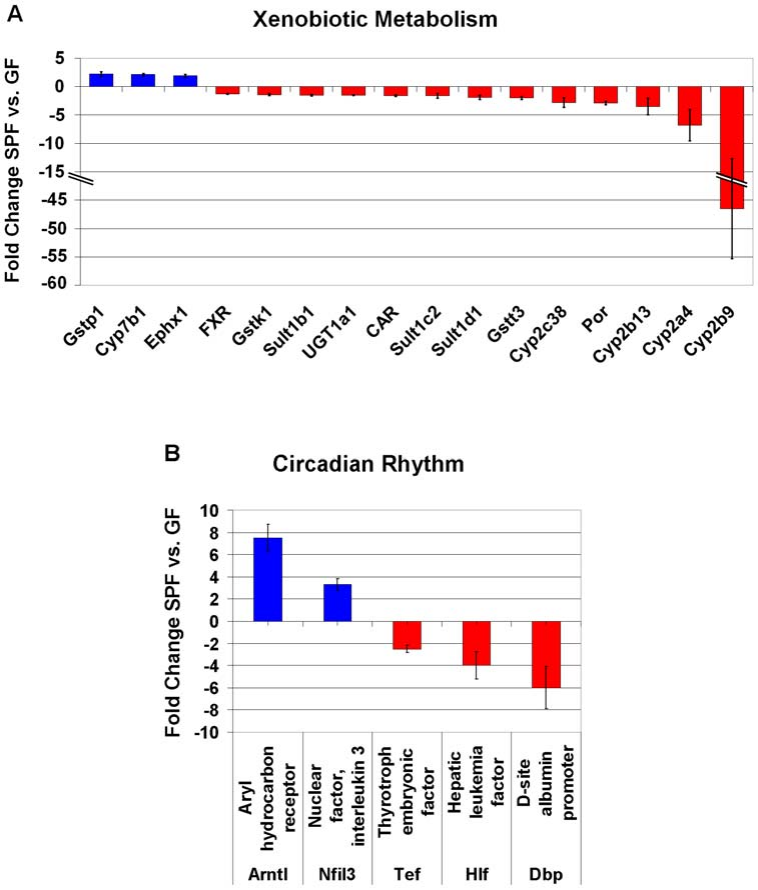
Selected differentially expressed genes in SPF compared to GF NMRI mice (n = 7 animals/group). Fold changes of genes in the categories (A) Xenobiotic Metabolism and (B) Circadian rhythm, are shown.

Gene expression in the liver was initially assessed on 60-mer oligo microarrays on RNA from GF and SPF male NRMI mice (n = 4/group; NCBI GEO platform accession GSE14689, according to MIAME guidelines). The genes that were significantly differentially expressed were selected for quantitative PCR-verification based on the following criteria: q<5% (false discovery rate), fold change (FC) of 2≤FC≥−2 and the presence of a human orthologue resulting in 380 genes. These selected genes were reanalyzed on TLDA or using individual qRT-PCR assays (n = 7 mice/group), and 112 genes were verified to be significantly differentially expressed (q<5%) in GF compared to SPF livers (Table S1). Using Ingenuity Pathway Analysis (IPA) we examined the relation between these 112 genes and the most significant canonical pathways or biological networks involved. As a result, the most abundant categories of differentially expressed genes are the endobiotic and the xenobiotic metabolism groups (Figure 1). Notably, biological network analysis shows that several of the genes modulated by the intestinal microbiota are regulated through a general xenobiotic regulator, the nuclear receptor CAR (Figure 2). CAR was 1.6-fold lower expressed in the SPF compared to GF mice, and several CAR-regulated genes were differentially expressed in the same direction. Some members of the cytochrome P450 family, involved in the metabolism of drugs, chemical carcinogens, and endogenous chemicals were also down-regulated in the SPF mice. Cyp2b9, a major P450 in the mouse liver was 46.5-fold lower expressed in the SPF compared to GF mice. Other P450s namely Cyp2a4, Cyp2b13, Cyp2c38 and Cyp4a14 were 6.8-fold, 3.5-fold, 2.8-fold and 8.4-fold lower expressed in SPF mice respectively. Interestingly, the P450 oxidoreductase (POR), the unique electron donor for all type II cytochrome P450 enzymes, was 2.9-fold lower expressed in the SPF animals compared to GF (Figure 3). The NR farnesoid-X receptor, FXR was also 1.3-fold lower expressed in the SPF compared to GF mice. Another group of genes that are expressed at lower levels in SPF mice are key regulatory genes in circadian rhythm, notably the PAR bZip genes, Dbp, Tef and Hlf, that were 6-fold, 2.5-fold and 4-fold lower in SPF compared to GF mice, respectively. A related gene, Nfil3 is 3.3-fold higher expressed in SPF mice. Finally, another gene of relevance to development and maintenance of neurons, Ntrk2, is 9.8-fold higher expressed in SPF mice.

### Effect of intestinal microbiota on liver function and size

**Figure 4:**
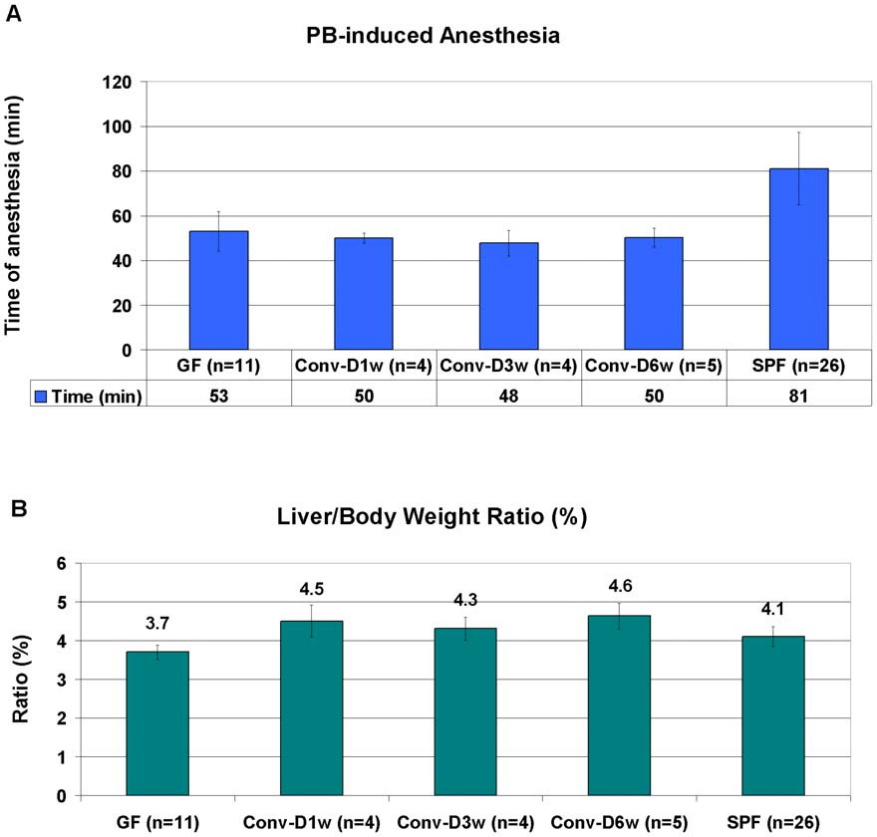
(A) Average time between loosing and regaining the righting reflex after i.p. injection (45 mg/kg body weight) of Pentobarbital and (B) Liver/Body weight ratio (%) in GF, Conv-D and SPF NMRI mice. GF: germ-free; Conv-D: conventionalized 1, 3 or 6 weeks; SPF: conventionally-raised specific pathogen free.

In order to address possible physiological consequences of the observed differences in gene expression, we studied the xenobiotic metabolic pathway of barbiturates and compared metabolism of pentobarbital in GF and SPF mice by monitoring their time of anesthesia. In line with the higher expression levels of CAR in GF mice, we observed that GF animals had a significantly shorter time of anesthesia as scored by the time between loosing and regaining the righting reflex after barbiturate injection compared to SPF animals (53 vs. 81 min, Figure 4, p<0.001, Student's t-test). Interestingly, in the conventionalized (Conv-D) groups, the barbiturate-induced anesthesia was similar to the GF group: 48 to 50 min (Figure 4).

We also examined liver weight in relation to body weight (liver/body weight ratio), given that this ratio is generally inversely correlated to xenobiotic metabolic capacity. This parameter was significantly lower in GF mice, 3.7% compared to 4.1% in SPF (p<0.001, Student's t-test, Figure 4). Surprisingly, the liver/body weight ratios for the Conv-D groups were actually higher than both SPF and GF; 4.5%, 4.3% and 4.6% after 1, 3, and 6 weeks conventionalization, respectively. However, the differences of all Conv-D groups compared to GF, and the Conv-D6w vs. SPF comparison reached statistical significance (Figure 4, p<0.001, Student's t-test). In conclusion, for the Conv-D mice the pentobarbital-induced anesthesia remained similar to the GF group, while the liver size in relation to body weight turned out to be more comparable to the SPF group.

## Discussion

In the present study, we connect the gut microbiota with liver gene expression profiles. The most abundant categories of the 112 differentially expressed genes are those involved in endobiotic and xenobiotic metabolism (Figure 1 and Table S1). Thus, we focus our discussion on key genes reported to have important liver functions.

Of particular interest was the observation of a network of modulated genes influenced by a key general xenobiotic regulator: the NR CAR. CAR was more highly expressed in the livers of GF compared to SPF mice, and several CAR-regulated genes were differentially expressed in the same direction. We have previously shown that CAR is also expressed at higher levels in GF colonic epithelium, when compared to SPF [16]. In addition, the CAR-regulated gene POR, coding for the P450 oxidoreductase, the unique electron donor for all type II cytochrome P450 enzymes, was more highly expressed in the GF animals compared to SPF. Currently, we can only speculate on the signals leading to the higher expression of CAR in the absence of an intestinal microbiota. However, it is known that levels of bile acids, steroid hormones and bilirubin are regulated by gut microbiota [9]–[12], [17]–[19]. In many cases, these cholesterol-derived metabolites in their unmodified form, act as ligands or activators of NRs in the liver to control the endogenous metabolism of the compounds. In the liver, bile acids, bilirubin and steroid hormones as well as xenobiotic compounds, activate CAR [13], [15], [20], [21]. Indeed, it has been known for decades that the intestinal microbiota influences the levels of bilirubin [18], [19], the composition and levels of bile acids [11], [12] and the amount of steroid hormones in feces and serum [9], [10], [12], [17]. GF animals also have reduced fecal secretion of bile acids and neutral steroids and increased levels of cholesterol in the liver and serum [17]. Gustafsson *et al*. tested the microsomal metabolism of GF and Conv rats by cholesterol feeding [12]. This leads to steroid and bile acid profiles in conventional animals that are similar to that of GF. Interestingly, the high hydroxylase activities characteristic of the GF rat was only slightly decreased as long as 56 days after conventionalization. Our results point to a constitutively higher expression of CAR in the absence of the intestinal microbiota in GF mice, likely because of the elevated levels of the CAR activators bilirubin, bile acids and steroid hormones, the relative contributions of these are so far unknown. This might be a result of the increased levels of cholesterol, and/or the direct biochemical effects of the microbiota on these compounds, leading to accumulation.

The increased basal level of CAR and its downstream target genes in GF animals was associated with significant effects on xenobiotic metabolism as assessed by challenge with pentobarbital. This challenge resulted in 35% shorter time of barbiturate-induced anesthesia in GF mice (53 min) compared to SPF (81 min), indicating a more efficient metabolism of the xenobiotic in GF animals. Other key regulators of metabolism that might be involved include the NRs farnesoid-X receptor FXR and pregnane-X receptor PXR. Interestingly, while the NR FXR was more highly expressed in the GF, compared to SPF mice, no significant difference in expression of PXR was detected indicating that the xenobiotic NRs involved may be differentially regulated by the gut flora.

Another group of genes of interest are the PAR bZip genes Dbp, Tef and Hlf. This group of genes are expressed at higher levels in GF mice and are reported to be key regulatory genes in circadian rhythm and xenobiotic metabolism [22], [23]. In contrast, the bZip transcription factor Nfil3, is higher expressed in SPF mice. This regulatory gene is known to be in the opposite phase to the rhythm of the PAR bZip genes in the liver, as well as in the hypothalamic suprachiasmatic nucleus (the mammalian circadian rhythm center) [24]. Furthermore, the PAR bZip genes and Nfil3 also share overlapping DNA-binding specificity [24]. Importantly, the PAR bZip genes control expression of two genes essential for xenobiotic metabolism: aminolevulinic acid synthase (ALAS1) and POR, with a 2.0-fold and 2.9-fold higher expression in GF mice respectively [22]. The PAR bZip genes also control the circadian expression rhythm of CAR [25]. Another important gene in circadian regulation, the aryl hydrocarbon receptor nuclear translocator like (arntl or BMAL1) is 7 fold lower expressed in GF mice. This gene has also been shown to be involved in regulation of xenobiotic metabolism [26]. Taken together, these data make it tempting to speculate on the possibility that the gut microbiota may impact on regulation of the peripheral circadian rhythm of xenobiotic metabolism, which opens up hitherto unsuspected lines of research.

An additional liver parameter that differed between GF and SPF animals was liver weight in relation to body weight, being significantly lower in GF mice. Our observed association between lower expression of POR and the three PAR bZip genes; Dbp, Tef and Hlf, and the higher liver/body weight ratio in SPF are consistent with results from hepatic POR null mice [27] and PAR bZip triple knockouts [23] where the liver/body weight ratios are increased, most likely because of accumulation of lipids. However, in CAR null animals, the ratio seems to be unaffected in untreated animals [21].

Another striking observation of our work was the almost 10-fold higher expression of Ntrk2, the gene encoding the neurotrophic tyrosine receptor kinase 2 TrkB, in SPF mice. The neurotrophins are growth factors that promote survival in development and maintenance of neurons. TrkB is a membrane-bound neurotrophin receptor that phosphorylates itself, and members of the MAPK pathway, after binding of its ligand; the brain-derived neurotrophin (Bdnf) [28], [29]. Interestingly, Ntrk2 or Bndf homozygous knockouts are not viable, but Bndf heterozygous knockouts, as well as conditional deletion of Bndf in the postnatal brain and hypomorphic mice expressing 24% of normal levels of TrkB all exhibit increased food intake and obesity [28], [30]–[32]. Intriguingly, in the brain, disruption of these molecules have also been shown to have cognitive effects such as hyperactivity, increased anxiety and increased locomotive activity [28].

A common approach to confirm microbiota involvement is to use conventionalization, i.e. to colonize GF mice with a complete microbiota (Conv-D) and compare them to conventionally-raised SPF mice. Previous reports indicate that conventionalization for up to 2 weeks renders Conv-D animals indistinguishable from animals conventionally-raised from birth [1]–[3], [5]. Thus, the physiological end-points in our experiments; liver size and xenobiotic metabolism, were tested in Conv-D mice 1 week, 3 weeks and 6 weeks after conventionalization. Already after one week, the liver weight increased so that the liver/body weight ratio was indistinguishable from SPF animals. This effect was even more pronounced after six weeks conventionalization, where the liver/body weight ratio was 13% higher than SPF, and as much as 20% higher than GF. Surprisingly, we were unable to render the rate of xenobiotic metabolism comparable to SPF animals even after conventionalization for 6 weeks. This indicates, as shown by B.E. Gustafsson for bile acids and steroids already in 1975, that the consistent reduction of microsomal metabolism after microbial colonization of the adult GF-born animal to the levels of rodents conventionally-raised from birth need considerable time, more than six weeks [12]. An alternative explanation is that while certain microbiota modulated phenomena such as liver weight, angiogenesis and obesity are subject to microbiota regulation throughout life; pathways such as regulation of xenobiotic metabolism may be fixed after a period early in postnatal life and hence can not be modulated later in life. Exposing young but not old GF mice to gut microbiota, showed that for the HPA (hypothalamic-pituitary-adrenal axis) system to become fully susceptible to inhibitory neural regulation, it must be exposed gut microbiota at an developmental stage when brain plasticity may still be preserved [33]. This data support the possibility of a constraint temporal window in development when gut microbiota can affect homeostasis.

Our results provide a novel mechanistic explanation for the effects of the microbiota on xenobiotic/drug metabolism in the liver. It is tempting to speculate that the effects on drug metabolism observed here extend to other drugs as well, which would potentially have immense influence on the general view of human variation of efficiency of xenobiotic metabolism by the cytochrome P450-system, an obstacle in pharmacology. Our findings will also have implications for the described correlation between xenobiotic metabolizing capacity and longevity [23], [34]–[36], a possible explanation for the longevity of GF rodents [37]

A curiosity is the described correlation between xenobiotic metabolizing capacity and longevity, e.g. in the long-lived Little mouse (homozygous for a missense mutation in the growth hormone releasing hormone receptor gene, *Ghrhr*) or in calorie-restricted mice, with upregulated xenobiotic response [34]. In the Little mouse, CAR and PXR are not involved in the upregulation of xenobiotic metabolism (except for Cyp2b10 and Cyp2C38), but the effect was attributed to increased levels of bile acids and signaling through FXR. GF mice have a longer lifespan than SPF, but the effects of calorie restriction were similar on GF and SPF mice [38] and rats [36], [37]. The general theory is that the accumulation of toxic metabolic by-products is a major contributor to ageing [36].

To summarize, the lack of intestinal bacteria in GF mice leads to excess amounts and accumulation of CAR-ligands such as bilirubin, bile acids and steroid hormones. This will maintain the downstream CAR target genes in an active mode and ensures a rapid and efficient turnover of the xenobiotic metabolic pathway. In turn, the PAR bZip genes are reported to act upstream of CAR thus connecting these two clusters of genes. Finally, our data seem to connect fundamental mammalian pathways such as xenobiotic metabolism, lipid and energy homeostasis, body weight, circadian rhythm and possibly longevity through regulation by the intestinal microbiota. Consequently, to target the gut microbiota with nutritional metabolites and tailor-made pre- and probiotics to modify drug metabolizing capacity is an interesting challenge for the future.

## Methods

### Animals and treatment

NMRI mice were maintained on a standard R36 chow (Lactamin, Sweden) in 12 hours light cycles. Male 9–12 weeks old mice were used. Animals were raised either under germ-free conditions (GF) in stainless steel isolators, or under conventional conditions in a specific pathogen-free environment (SPF). The GF status of the isolators was monitored weekly. A subset of the GF animals was colonized with a complete flora, a procedure termed conventionalization (Conv-D). The mice were sacrificed by cervical dislocation, the livers were harvested, weighed and stored in RNA later (Qiagen, Germany) at −80°C until RNA analysis, or flash-frozen in liquid nitrogen.

Pentobarbital (5-Ethyl-5-(1-methylbutyl)-2,4,6(1H,3H,5H)-pyrimidinetrione) was injected intraperitoneally, and the time between loosing and subsequently retrieving the righting reflex was assessed. Eleven GF and 26 SPF NMRI mice were tested. The body- and liver weights were recorded post mortem. To assess the time scale of the effect of the flora on the pentobarbital anesthesia and liver size, GF mice were Conv-D for 1, 3, or 6 weeks (n = 4–5/group) before pentobarbital-induced anesthesia and liver/body-weight ratio were measured. Animal husbandry was in accordance with institutional guidelines at Karolinska Institutet and all animal experiments were approved by the animal ethics committee north at Karolinska Institutet, Stockholm, Sweden.

### RNA extraction

Total RNA was extracted using Qiagen RNeasy midi kit, according to the manufacturer's instructions and was stored at −80°C. RNA concentration and purity were determined using Nanodrop at wavelength 260/280 nm and RNA integrity was ensured using agarose gel electrophoresis.

### TaqMan Low Density Array

Microarray analysis was carried out on the liver samples of SPF and GF mice by using the Compugen mouse 22K oligonucleotide arrays (NCBI tracking system #15583764). Transcript levels for 380 genes and the reference gene 18S rRNA (Table S1) were analyzed by a Taqman low-density array (TLDA) to verify the microarray results. Total RNA (1 µg) (n = 7 mice/group) was reverse transcribed into cDNA by using a High-Capacity cDNA Archive Kit (Applied Biosystems, Foster City, CA). The reverse transcriptase reaction was performed at 25°C for 10 min and then 37°C for 2 h, followed by 85°C for 5 s. Each cDNA sample (100 µg/50 µl) was added to an equal volume of 2x Taqman Universal PCR Master Mix (Applied Biosystems). The mixture was then transferred into a loading port on a TLDA card (Applied Biosystems). The card was centrifuged twice for 1 minute each at 1,200 rpm to distribute the PCR mix from the loading port into the wells of the card. The card was then sealed and PCR amplification was performed using an Applied Biosystems Prism 7900HT sequence detection system. Thermal cycler conditions were as follows: 50°C for 2 min with 100% ramping, 94.5°C for 10 min with 100% ramping, 40 cycles of 97°C for 30 s with 50% ramping, and 59.7°C for 1 min with 100% ramping. The data was analyzed using the SDS2.2 software (Applied Biosystems) where baseline and threshold settings were automatically adjusted. The SAM (Statistical Analysis of Microarrays) statistical method was used to select differentially expressed genes. Genes were selected based on the q-value less than 5%.

### Gene ontology clustering

SAM-generated differentially expressed gene lists were analyzed for gene ontology information by using Ingenuity Pathways Analysis (IPA) software (Ingenuity® Systems, www.ingenuity.com), which looks for both known canonical pathways and networks of genes. For canonical pathways, the significance of the association was measured in two ways: (i) by the ratio of the number of genes from the data set that map to the pathway divided by the total number of genes in that pathway and (ii) by using the Fisher exact test to calculate a *P* value determining the probability that the association between the genes in the data set and the canonical pathway is explained by chance alone. For the networks, Ingenuity Pathways analysis computes a score for each network according to the fit of the user's set of significant genes. The score is derived from the likelihood that the focus genes in a network are together due to chance alone. A score of 2 is equivalent to a *P* value of 0.01.

## Supporting Information

Table S1 List of genes tested with TLDA, including differentially regulated genes in the livers of SPF compared to GF NMRI mice.
(0.09 MB XLS)
